# Design and Analysis of a True Random Number Generator Based on GSR Signals for Body Sensor Networks

**DOI:** 10.3390/s19092033

**Published:** 2019-04-30

**Authors:** Carmen Camara, Honorio Martín, Pedro Peris-Lopez, Muawya Aldalaien

**Affiliations:** 1 Department of Computer Science, University Carlos III of Madrid, 28911 Leganes, Spain; pperis@inf.uc3m.es; 2 Department of Electronic Technology, University Carlos III of Madrid, 28911 Leganes, Spain; hmartin@ing.uc3m.es; 3 Higher Colleges of Technology, Abu Dhabi Women’s College, Abu Dhabi 41012, United Arab Emirates; maldalaien@hct.ac.ae

**Keywords:** Galvanic Skin Response (GSR), entropy, randomness, Random Number Generators (RNG), Hilbert transform

## Abstract

Today, medical equipment or general-purpose devices such as smart-watches or smart-textiles can acquire a person’s vital signs. Regardless of the type of device and its purpose, they are all equipped with one or more sensors and often have wireless connectivity. Due to the transmission of sensitive data through the insecure radio channel and the need to ensure exclusive access to authorised entities, security mechanisms and cryptographic primitives must be incorporated onboard these devices. Random number generators are one such necessary cryptographic primitive. Motivated by this, we propose a True Random Number Generator (TRNG) that makes use of the GSR signal measured by a sensor on the body. After an exhaustive analysis of both the entropy source and the randomness of the output, we can conclude that the output generated by the proposed TRNG behaves as that produced by a random variable. Besides, and in comparison with the previous proposals, the performance offered is much higher than that of the earlier works.

## 1. Introduction

The proliferation of wearable sensors has meant that medical environments are not the only ones in which the acquisition of vital signs can occur [1]. For instance, there are a large number of smart-watches (or sports watches) that monitor several of our physiological signs throughout our daily lives, and even smart-textiles that have one or more integrated sensors have appeared on the market [2]. Concerning the measured signal, there is a wide variety ranging from signals related to the brain (e.g., Electroencephalogram (ECG)) through signals linked to the heart (e.g., Electrocardiogram (ECG) or Photoplethysmogram (PPG)) to signals related to emotions (e.g., Galvanic skin response (GSR)). Sensors do not usually work in isolation but form a network. When we refer to sensors that are in (e.g., a pacemaker or a neurostimulator) or around (e.g., an insulin pump or a sport-watch) the body, this type of network is named Wireless Body Area Network (WBAN) [3,4]. Body Sensor Network (BSN) or Medical Body Area Network (MBAN) are other names given to these networks [5,6]. Apart from the sensors, there is a central element called the gateway—a smart-phone usually implements the latter. Currently, the sensors do not communicate directly with each other (shortly this may happen), but all connections pass through the gateway. It is also the gateway that provides connectivity to the Internet [7].

### 1.1. Related Work

In the context of cybersecurity, vital signs have proved to be very useful in recent years. Biometrics solutions based on ECG [8,9] or EEG signals [10,11] have been proposed for authentication purposes. Some authors have even studied its feasibility (ECG [12,13] or EEG [14,15]) in the context of continuous authentication—the verifier validates the credentials at regular intervals, ideally at every instant. The key distribution problem between two devices (e.g., two ECG sensors [16]) has also attracted the attention of some researchers. In detail, in these solutions, each sensor derives the shared key from the acquired physiological signal, preventing the sensors from sharing any information beforehand [17,18]. In addition, the extraction of randomness from physiological signals has been recently scrutinised (e.g., ECG [19,20], EEG [21,22] and EMG [23]).

Regarding MBAN, the security of Implantable Medical Devices (IMDs) has attracted the attention of many researchers [24,25]. Even the FDA has alerted users of some vulnerabilities in commercial IMDs [26]. The proposed solutions to increase the security level of these critical devices are very diverse [27]. Some authors propose the usage of logs for auditing purposes [28] or the use of an external device that filters the messages sent to the implant [29]. The use of biometrics solutions, such as those based on fingerprints or iris, has been recently proposed [30,31]. Classical approaches based on symmetric [32,33], asymmetric [34,35] or hybrid ones [36] have been also suggested. Some authors have found interesting the combination of authentication schemes and distance bounding protocols [37]. Besides, some new research work focuses on the key distribution problem [16,38] and how to extract randomness from the signal acquired by the implant (mainly cardiac implants) [39,40,41].

The use of reliable Random Number Generators (RNGs) is crucial in security systems. Even well-known modern cryptographic solutions, such as the RSA private keys of HTTPS hosts, may have been compromised due to failures in the generation of nonces on networked devices [42]. When computational algorithms are used to generate random numbers, they are called Pseudorandom Number Generators (PRNGs). PRNGs depend on an initial value, called seed or key, and the outputted bitstream behaves as a random variable [43,44]. Alternatively, we can use physical phenomena with high entropy (e.g., atmospheric noise or decay of a radioactive source) as a source of randomness. This type of generators is called True Random Number Generators (TRNGs) [45,46].

In this article, we propose the design of a TRNG based on the GSR signal. As explained below, the parasympathetic nervous system controls the GSR signal. Therefore, instead of a physical phenomenon, we exploit a physiological signal that we carry with us—each user is the bearer of her or his random number generator. Besides, the GSR signal cannot be self-controlled, which prevents an attacker (or the carrier) from causing misbehaviour in the signal. As far as we know, Tuncer and Kaya [23] reported the only work close to our proposal that analyses the use of various biosignals, including the GSR signal, as a source for a random number generator. Unfortunately, in relation to the GSR signal, the proposal has been validated with only 12 subjects (much lower than 86 in our case; see Section 2.1) and the throughput (64 bits per second in the best case) is far from that offered by our proposal (1024 bits/s), as shown in the next sections.

### 1.2. Galvanic Skin Response

The electrical conductivity of our skin undergoes subtle alterations every time we are emotionally aroused. The Galvanic Skin Response (GSR)—also known as Electrodermal Activity (EDA) or Skin Conductivity (SC)—is often used, because of its sensitivity, to measure these variations. Therefore, the GSR measures the changes in the electrical characteristics of the skin. Humans have between two and five million sweat glands; men and women have the same number of glands, but male glands secrete five times more in size and volume [47]. Likewise, sweating is triggered when we are exposed to emotional stimulation. Perspiration through skin pores makes changes in the balance of positive and negative ions in the secreted fluid. As a result, we can observe changes in skin conductance. Note that an increase in skin conductivity means a decrease in skin resistance.

The Autonomic Nervous System (ANS), which forms with the Somatic Nervous System (SNS) the Peripheral Nervous System (PNS), controls the functioning of many organs, muscles, and glands [48]. In detail, this regulation (proper behaviour of our body) is achieved by impulses from the brain (and/or spinal cord) and generated by autonomous neurons. Sweet glands are part of the glands mentioned above. In detail, sweating is driven and balanced by the ANS, and we cannot consciously control it. The ANS consists of the parasympathetic and the sympathetic nervous system [49]. The former is responsible for “rest and digest”. Decreased heart rate, decreased sweating, or decreased blood pressure are some effects of its activation. The latter is responsible for the body’s “fight or flight” reaction. That is, it helps to protect the body and is involved in functions such as pupils dilatation, increased heart rate or sweating [50]. Therefore, both systems are complementary to each other.

**Figure 1:**
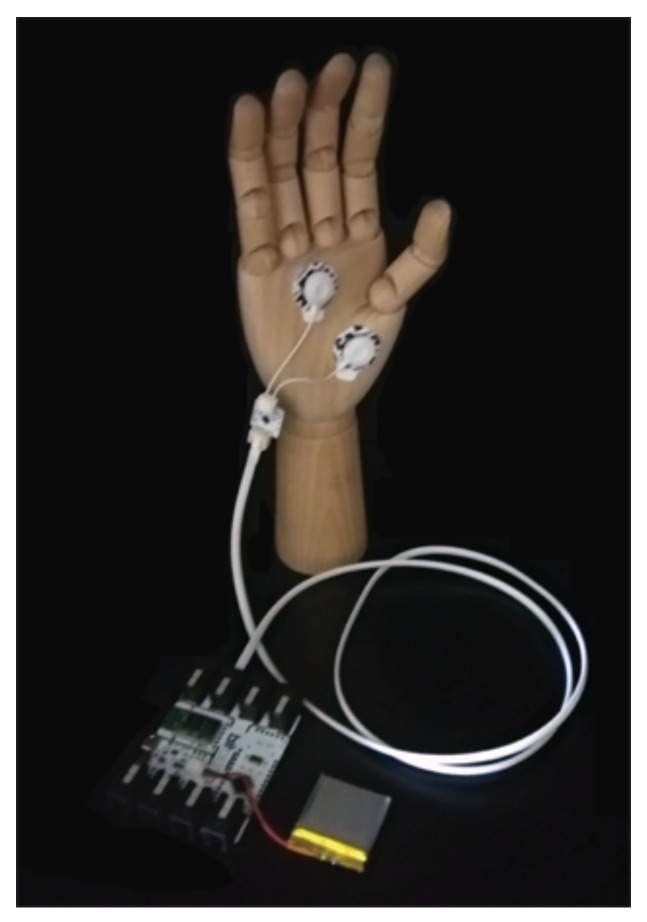
Electrodes placement for GSR acquisition.

The recording of the GSR signal is non-invasive, and we only need two electrodes for its acquisition. Three are the most common placements: (1) index and middle fingers; (2) left and right side of palm; and (3) foot. In the market, we can find low-cost hardware platforms (e.g., BITalino or Libelium e-Health platform [51]) for the acquisition of biosignals. In Figure 1, we show an example of the electrode placement using the Bitalino platform for the signal acquisition. In detail, the exosomatic method with a small constant voltage is the most common approach to measure the GSR signal. The skin conductance (1/resistance) values are determined by measuring the changes in the current flow between the two electrodes, as the voltage is constant [52].

## 2. Methods and Materials

### 2.1. Dataset Description

The randomness test batteries (e.g., DIEHARD [53] and NIST [54]) commonly used to verify the randomness quality of a random number generator require files of several tens of megabytes. For this reason, the GSR signals used in this study come from three well-known datasets:

1. The Affective Pacman (AffPac) dataset [55]. Twelve healthy users (aged 27 ± 3.9; 25% female) participated in the experiment. Several physiological signals were recorded simultaneously, including EEG, EOG and GSR signals.
2. DEAP dataset [56]. Thirty-two healthy participants (aged 28 ± 9; 50% female) volunteered for the experiment. The subjects watched several music videos while the physiological signals (e.g., EEG and GSR) were acquired.
3. AMIGOS dataset [57]. Forty healthy users participated in the experiment (aged 30.5 ± 9.5; 32.5% female). The participants watched short (16) and long (4) emotional videos. Three neuro-physiological signals (i.e., EEG, ECG and GSR signals) were recorded using wearable sensors. In our experiments, we discarded three files (subjects) because of their short length.

Note that we discarded the acquisition of our own GSR signals (e.g., using the Bitlanino platform) because, for our experimentation, we needed signals from many subjects and at the same time very extensive in time. As mentioned, in our experiments, we used signals from three datasets forming a total of 82 individuals (aged 28.5 ± 7.5; 35.8% female). Since no individuals present any severe pathology, we can then discard any bias in the output bits generated by the proposed TRNG. Furthermore, the signal acquisition process guaranteed that the GSR signals of the subjects in the dataset are statistically independent.

### 2.2. Methods

**Figure 2:**
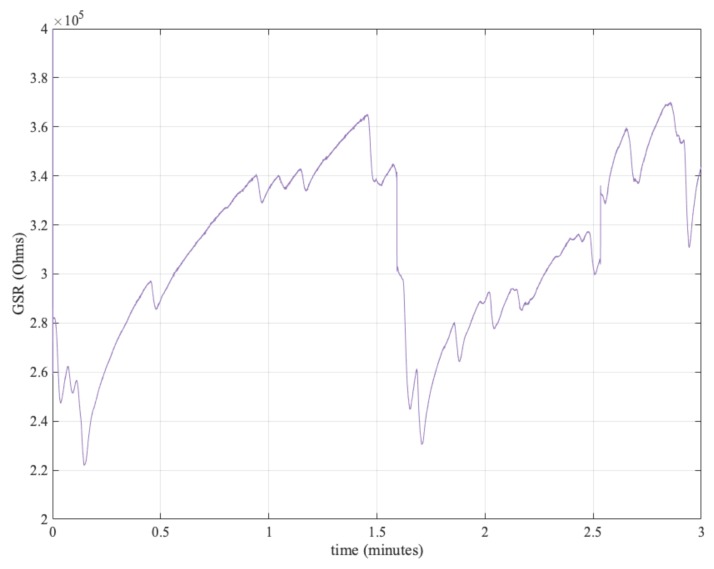
GSR signal.

In our experiments, we focused exclusively on the GSR signal. We aimed to validate the hypothesis we can extract randomness from this vital signal. The proposed procedure is summarised in Algorithm 1 and explained below. First, for the GSR signal pre-processing, we followed a similar approach with all three datasets. As a first step, the data were down-sampled to 128 Hz. Then, a low-pass filter with 60 Hz cut-off frequency was applied. As an illustrative example, Figure 2 shows three minutes of a GSR signal.
**Algorithm 1** GSR-TRNG.  1:  **procedure**
Pre-processing($GSR^{raw}$)  2:  Down-sampling to 128 Hz  3:  Low-pass filter ([0−60Hz])  4:  **procedure**
GetEntropy($GSR^{cleaned}$)  5:  Split $GSR^{cleaned}$ into N-seconds GSR-windows (N=4 in our experiments)  6:  **for**
each GSR-window($x^{\left(j\right)}\left(t\right)$)
**do**  7:    Hilbert Transform:
$y^{\left(j\right)}\left(t\right)=h\left(t\right)*x^{\left(j\right)}\left(t\right)$  8:    Entropy Extraction:
$g^{\left(j\right)}{\left(t\right)}_{\left(0,\ldots ,7\right)}=uint8\left(\left(uint32\left(abs\left(y^{\left(j\right)}\left(t\right)*10^{2}\right)\right)\right)>>24\right)$

After cleaning the GSR signal, we needed to extract randomness numbers from it. For this, we divided the GSR signal into windows of N=4 seconds to be able to capture some variability in the signal—we fixed the size of the window by experimenting after analysing an extensive set of possible values. Secondly, we computed the Hilbert transform for each window. Hilbert transform can be interpreted as an all-pass filter in which all positive/negative frequencies are sifted −90/90 degrees, respectively. Mathematically, the Hilbert transform of a real, continuous-time signal is given by:

(1) y(t)=h(t)∗x(t)

where h(t) represents the Hilbert transform kernel ($h\left(t\right)=\frac{1}{\pi t}$, t∈(−∞,∞)).

Finally, we extracted random bits from the Hilbert transform values. Mainly, we used an entropy extraction algorithm for this purpose. More precisely, using an accuracy of six decimal places, each value was converted to a 32-bit unsigned integer value, and then a byte was extracted from the Least Significant Bits (LSBs). It means that the proposed TRNG can generate 8×fs bits per second, with fs being the sampling rate used. The use of the LSBs is motivated by the fact that it is in these positions where there is more variability (randomness, formally stated) as confirmed by the results presented in the following sections. Mathematically, the extraction of random bits can be expressed as:

(2) $$g{\left(t\right)}_{\left(0,\ldots ,7\right)}=uint8\left(\left(uint32\left(abs\left(y\left(t\right)*10^{2}\right)\right)\right)>>24\right)$$

Once we specified the randomness extraction algorithm, we needed to assess the quality of the random numbers generated. For this purpose, we used the datasets introduced in Section 2.1. The reader can consult the following section for an in-depth security analysis of the proposed TRNG.

## 3. Results

We analysed the proposal from two perspectives. Firstly, the quality of the entropy source was studied, using the NIST SP 800-90B recommendation [58]. Secondly, the randomness of the random numbers generated was examined using well-known batteries of tests, such as DIEHARDER [53] and NIST [54].

### 3.1. Source Entropy Analysis

A cryptographic Random Bit Generator (RBG) is composed of three components: (1) an entropy source; (2) an algorithm responsible of storing and providing bits to the target application, and (3) the procedure for combining the two first components. In a nutshell, the entropy source model consists of an analogue noise source (in our case, the GSR signal, which is first cleaned with the procedure Pre-Processing in Algorithm 1) and a digitisation algorithm (procedure GetEntropy specified in Algorithm 1 and defined by Equations (1) and (2)).

For testing the entropy of RBGs, the NIST SP 800-90B recommendation proposes ten estimators, including the Markov and LZ78Y estimate among others for calculating the min-entropy [58]. The final estimation is the minimum value of all these tests. A file of 25 million 1s and 0s was generated using the third dataset to evaluate the entropy quality of the GSR signal. In most tests (see Table 1), the entropy value was close to the optimal (1) and even for the worst case remained very high (0.935). In this particular case, the t-tuple test sets the min-entropy value. This test evaluates the frequency of pairs, triples, and so on, and estimates the entropy per sample based on these frequencies [58]. From all the above, fortunately, we can conclude that the GSR signal together with the proposed digitisation algorithm seemed appropriate for cryptographic solutions.

In some occasions, the estimation of the entropy calculated on a very long sequence can produce an overestimation of the entropy—correlated sequences might be generated after a restart. If this is the case, the attacker could cause multiple restarts of the entropy source to generate an advantageous situation for her/him. The “restart” test is defined in the NIST SP 800-90B specification to evaluate this issue. As for generating data for this test, the GSR source was restarted $10^{3}$ times, and then we recorded $10^{3}$ consecutive values. In our case, we used the third dataset, in which the subjects were shown 20 different videos. Therefore, in our experiments, the reset of the physiological signal was simulated by exposing the subject to a different stimulation (video). Furthermore, to be even more confident, we repeated the test five times (i.e., from File-1 to File-5). As shown in Table 2, the five tests were passed successfully and confirmed that 0.94 was not an overestimate for the min-entropy.

### 3.2. Randomness Analysis

**Figure 3:**
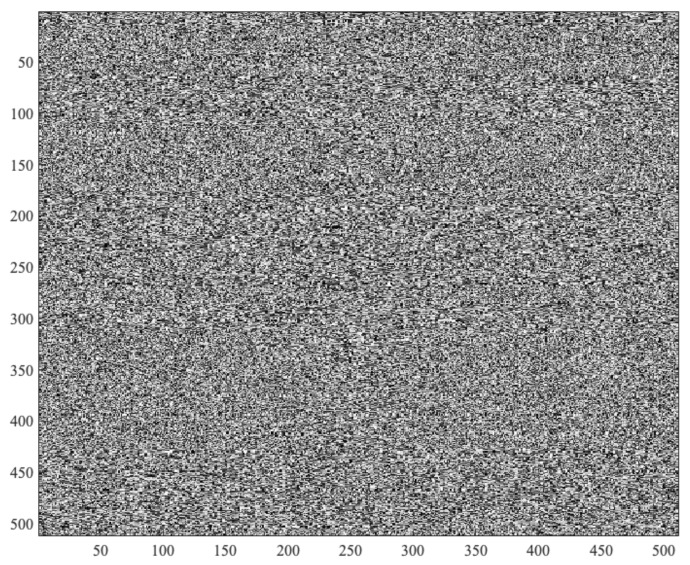
Random numbers generated by the proposed GSR-RNG.

In Algorithm 1, we included an entropy distillation process (Procedure GetEntropy) to produce randomness. After the entropy analysis, we needed to assess the randomness quality of the bits generated by the GSR-TRNG. For a first visual inspection in Figure 3, we show an 8-bit grey scale image (512 × 512) of values generated by our TRNG. No anomalous patterns were detected, and the image behaves as the one generated by any other strong cryptographic random number generator. Several test batteries are commonly used (ENT [59], DIEHARDER [53] and NIST [54]) to analyse the randomness in depth. These tests require an input file of several hundred million bits. In our particular case, we generated a file of 30 MBytes by joining the GSR signals (signals of 84 subjects in total) of the three datasets introduced in Section 2.1.

ENT suite [59], which is not intended for cryptographic applications, is one of the test batteries usually used first to discard weak or faulty generators without the need for additional testing. Table 3 shows the results after analysing the 30 MByte file mentioned above. The entropy and compression results indicate that the file was extremely dense in terms of information (randomness). As for the chi-square test, which is very sensitive to detect weak generators, the results show no g suspicion of being not random. The arithmetic mean value confirmed that the proportion of ones and zeros were equal (i.e., there was no bias in the output). The serial correlation coefficient showed the high unpredictability of the bitstream—there was a low dependence between a particular bit and its predecessors.

**Figure 4:**
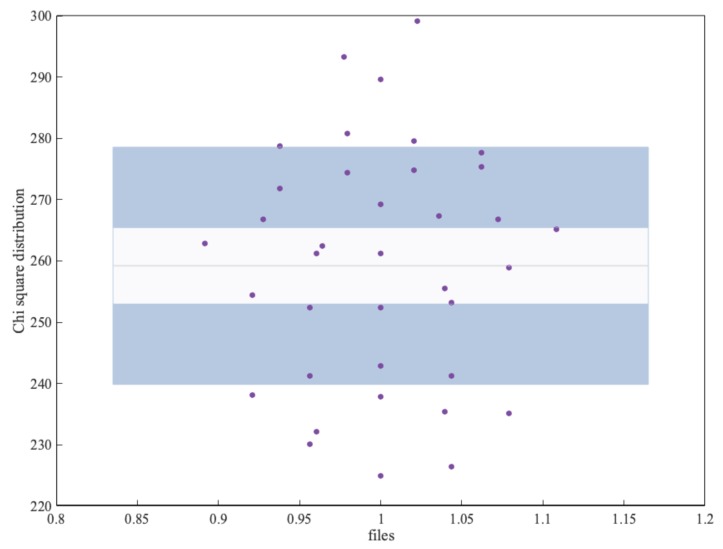
Bias analysis.

To analyse whether there were no biases in the behaviour of each subject’s signals, we performed an additional experiment by analysing them separately. Using the signal of the 37 subjects of the AMIGOS dataset, we generated a binary file of 800 KB for each of the subjects. Each of these files was analysed with the ENT suite. Figure 4 shows the result of the chi-square test. As shown in the figure, most values were within the optimal value (256) and ± the standard deviation. We can, therefore, conclude that the different subjects behaved similarly. In other words, there were no significant differences between the bitstreams generated from the different GSR signals corresponding to each subject.

DIEHARDER [53] (a modern version of the Diehard battery), and NIST [54] are much more demanding test batteries than ENT. NIST has been designed to test RNGs that are devoted to cybersecurity solutions. DIEHARD consists of 15 test and the results obtained are summarised with a *p*-value in Table 4a. In detail, all tests were within the interval [0.025–0.975]—note that, due to a large number of *p*-values calculated, it would not be uncommon for some of them to be outside this range. Apart from being distributed within the interval mentioned above (0.05 of significance level), the critical point to consider the file under analysis random is that these *p*-values must follow a uniform distribution. We tested this hypothesis using a Kolgomorov–Smirnov test, which returned a decision that the *p*-values come from a uniform distribution at the 5% of the significance level. Therefore, we can conclude (95% of confidence) that there were no bad behaviours in the analysed bitstream (30 MByte file) and that all the DIEHARD tests were successfully passed. As mentioned above, NIST is often used in the context of cybersecurity and for formal verification of RNG designs. The NIST suite is made up of 15 tests, which examine bits, m-bit blocks or m-bit parts. Regarding the interpretation of the results, the first value corresponds with the *p*-value calculated for uniformity testing with the *p*-values obtained with a given test; the values in brackets represent the proportion of tests passing the corresponding test. The following equation gives the minimum number of tests (except for the random excursion test) that must be passed for each test:

(3) $$mpr=\left(1-\alpha \right)-3*sqrt\left(\frac{\alpha *(1-\alpha )}{k}\right)$$

being (1−α) the significance level and *K* the number of sequences tested. In our particular case, α=0.01 and K=100, thus the minimum pass rate was 96. From the results in Table 4b, all the tests passed the uniformity test (*p*-values in the interval 0.01–0.99; α=0.01) and the proportion test was above the mentioned threshold (mpr=96). Furthermore, the Kolgomorov–Smirnov confirmed the uniformity of all *p*-values (15 tests) with 1% of significance level. From all this, we can conclude that the bits generated by the TRNG based on GSR signals behaved as a random variable.

**Figure 5:**
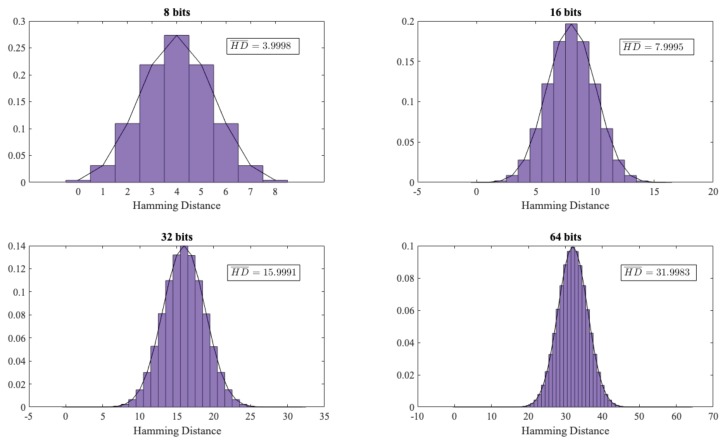
Hamming distance distribution.

As an additional experiment, we analysed whether there was any relationship between the random numbers generated by each user (GSR signal). If this were the case, it would be very advantageous for an attacker, since s/he could exploit the knowledge of a GSR signal (e.g., User-A) and predict the values of another signal (e.g., User-B). To assess this, using the 38 users of Dataset 3, we created a file of 800 KB. Next, we grouped the data of each file in words of different sizes (m={8,16,32,64}). For each of these word sizes, we computed the hamming distance between all the dataset pair combinations ($C_{38,2}$). We show the results obtained in Figure 5.

If there is no relation between the users (GSR signals), the calculated Hamming distance should follow a binomial distribution (p(X=k)=mkpk(1−p)n−k; E(X)=m∗p and $\sigma^{2}=n*p*\left(1-p\right)$) being *m* the size of the words and p=1/2 as the zeros and the ones are equally likely). In our experiments (see Figure 5), as expected, the experimental values were almost identical to the theoretical ones (i.e., a hamming distance of 4, 8, 16 and 32, respectively). Therefore, the advantage of an adversary of predicting the values of a user using the knowledge of other users’ signals was zero.

**Figure 6:**
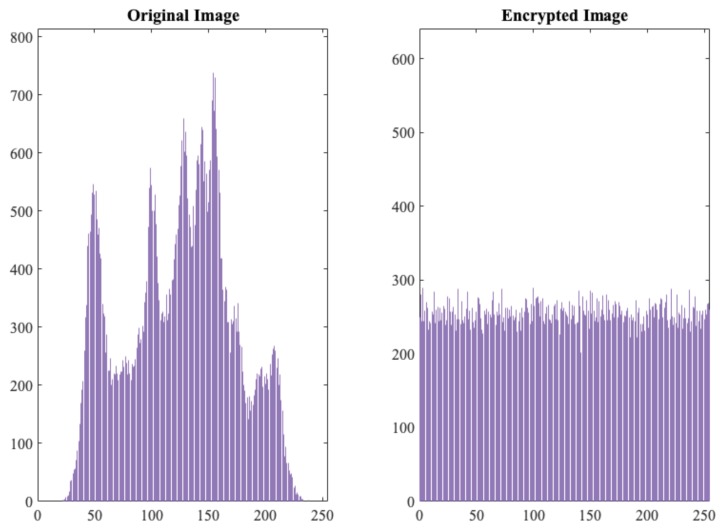
Original and encrypted statistical histograms.

Apart from the randomness tests, and as a final test, we analysed the TRNG as if it were used as a generator of a ciphering sequence (*s*) to encrypt a plain-text (*m*): c=E(s,m)=s⊕m. In particular, using this approach, five different images (256 × 256 grayscale images), chosen randomly from the Internet, were used as inputs for the experiment. As for the ciphering sequence, bits were grouped in bytes and then regrouped into a matrix of the same size as the inputs images. As a first glance, Figure 6 shows the histogram of one of the tested images and its histogram after encryption. As expected, the encryption made the histogram uniform. Note that, if *s* (image with random values) follows a uniform distribution, and *s* and *m* are chosen independently of each other, the resulting value is uniformly distributed, since we combine them with the bitwise operation. This uniform distribution at the output makes it impossible for an attacker to extract any information from the original plaintext (image from the Internet in our example). Nowadays, NPCR and UACI tests are used to evaluate the strength of an image encryption technique against differential attacks [60]. In short, the first assesses the number of changing pixels and the second evaluates the changes in intensity, in both cases, between two encrypted images when the two plain images differ by one bit. In Table 5, we summarise the results of these test for the five examined images. Considering the thresholds given in [61], NPCR and UACI tests passed successfully at 0.05 significance level (i.e., $NPRC_{0.05}\geq 99.5693\%$ and $33.284\%\leq UACI_{0.005}\leq 33.6447\%$).

## 4. Discussion and Conclusions

Today, there are many devices that monitor vital signs. These devices can be medical devices such as pacemakers or insulin pumps or general purpose devices such as sports watches or smart clothing with sensors. In any case, we have devices equipped with one or several sensors that transmit the acquired information (in most cases, wirelessly) to a central device. Although no one doubts the benefits of constant monitoring of our physiological parameters, access to these data only to authorised entities and their protection when transmitted through an insecure channel (mainly the radio channel) should be guaranteed from the design phase. Random number generators play a critical role in the design of cryptographic solutions for this purpose. Motivated by this fact, in this article, we have proposed a TRNG that benefits from a vital signal that is already being monitored by a sensor on the body. In particular, we have studied how to design a random number generator based on the GSR signal. Both the entropy source and the output randomness analysis confirm that the generated bitstreams behave as a random variable.

As shown in Algorithm 1, for the extraction of the randomness (Procedure GetEntropy), the Hilbert transform is used, which is usually used to construct the Analytic signal. Mathematically, given a signal x(t) and its Hilbert transform y(t), it is defined by $x_{A}\left(t\right)=x\left(t\right)+jy\left(t\right)$. In our particular case, we use only the imaginary part of the analytic signal that corresponds to the Hilbert transform itself. The reader may be tempted to think that the extraction of the entropy could be done from the signal itself (without any transformation). However, this was the first approach that we tested, and, although the output is entropic, a simple test such as the chi-square (ENT suite) clearly shows how the bits generated are non-random. Therefore, the use of Hilbert’s transform is justified. Note that the procedure for extracting random bits (see Equation (2) in Section 2.2) also plays a crucial role in our proposed TRNG.

In general terms, three elements are the main components of a TRNG: (1) noise source (GSR signal in our case); (2) digitisation algorithm; and (3) post-processing procedure (optional). In our case, we only have the first two elements since we consider that post-processing is not necessary. Among the most common post-processing techniques are bitwise XOR operations, Von Neumann algorithm or even the use of a hash function [62,63]. The use of these techniques is mandatory when the quality (randomness) of the output is not yet the desired. As shown in the in-depth analysis of the randomness (see Section 3.2), our generator successfully passes all the test batteries, and that is why our proposal dispenses with this stage.

A key parameter about any primitive cryptography is its performance. In the case of random number generators, high or moderately high throughput may be necessary for many applications. The proposed TRNG can generate 1024 bits per second (i.e., 8×fs=8×128). This performance is far superior to that achieved by other random number generators using biosignals. In this context, the cardiac signal is the most studied physiological signal for this purpose. Solutions based on Interpulse Interval (IPI) values can generate between 2 and 14 bits per second [38,64], which is far below our performance. Even modern solutions based on the wavelet transform offer a through three times lower [19]. Concerning the GSR signal and the recently proposed TRNG [23], its throughput is 16 times lower at best than that of our approach. We can conclude from all this that our proposal offers excellent performance to be used in cybersecurity solutions.

As shown in this article, a new generation of TRNGs based on our vital signs can be designed. Apart from the GSR signal, and cardiac signals, other signals, such a the electrical activity of the brain (e.g., electroencephalogram) or the skeletal muscles (e.g., electromyogram) could be employed. Even for highly demanding applications, the combined use of various signals could give excellent results. As a conclusion, we can state that just as we still have much to learn from the human body within medicine, the use of the body is even less explored for cybersecurity tasks. In addition, it is worth mentioning that the use of sensors, integrated into a wide variety of devices, plays a critical role in the acquisition of the signal at stake. 

## Figures and Tables

**Table 1:** Min-entropy results (NIST SP 800-90B Suite).

Method	Min-Entropy
Most Common Value Estimate	0.99876
Collision Estimate	0.966577
Markov Estimate	0.999052
Compression Estimate	1
t-Tuple Estimate	0.935861
LRS Estimate	0.965143
MultiMCW Prediction Estimate:	0.999605
Lag Prediction Estimate	0.999152
MultiMMC Prediction Estimate	0.998977
LZ78Y Prediction Estimate	0.998780
**Overall estimation**	0.935861

**Table 2:** Restart tests (NIST SP 800-90B Suite).

File ID	Result
File-1	Pass
File-2	Pass
File-3	Pass
File-4	Pass
File-5	Pass
**Final min-entropy estimation**	**0.94**

**Table 3:** ENT results.

Entropy	7.999994
Optimum compression	0%
Chi square	235.33 (80.64%)
Arithmetic mean value	127.4990
Monte Carlo π value	3.143071846 (error 0.05%)
Serial correlation coefficient	−0.000129

**Table 4:** DIEHARD and NIST Results.

*(a) DIEHARD Results*	
Birthdays	0.1079
OPERM5	0.1265
32x32 Binary Rank	0.5070
6x8 Binary Rank	0.6194
Bitstream	0.1318
OPSO	0.0386
OQSO	0.1792
DNA	0.1792
Count the 1s (stream)	0.9853
Count the 1s Test (byte)	0.2096
Parking Lot	0.0667
Minimum Distance	0.5923
(2d Circle)	
3d Sphere	0.9626
(Minimum Distance)	
Squeeze Test	0.8645
Sum Test	0.0340
Runs	0.2381 (up)
	0.6902 (down)
Craps	0.5847 (wins)
	0.3163 (throws)
*(b) NIST Results*	
Frequency	0.7792 (98/100)
Block Frequency	0.6787 (99/100)
Cumulative Sums	0.2974 (2/2)
	(99/100)
Runs	0.2368 (98/100)
Longest Run	0.7197 (100/100)
Rank	0.3345 (98/100)
FFT	0.8831 (99/100)
Non-Overlapping	0.5181 (148/149)
Template	(>99/100)
Overlapping Template	0.5749 (100/100)
Universal	0.3838 (99/100)
Approximate Entropy	0.0909 (100/100)
Random Excursions	0.6781 (8/8)
	(>61/62)
Random Excursions	0.5799 (18/18)
Variant	(>36/37)
Serial	0.8188 (2/2)
	(>99/100)
Linear Complexity	0.1296 (100/100)

**Table 5:** NPCR and UACI randomness tests.

	NPCR	UACI
File-1	99.6139%	33.6028%
File-2	99.6185%	33.6315%
File-3	99.5911%	33.2750%
File-4	99.6124%	33.4287%
File-5	99.6139%	33.4694%
**Optimal value** (256 × 256) [61]	$NPCR_{0.05}\geq 99.5693\%$	$33.2824\%\leq UACI_{0.005}\leq 33.6447\%$
	$NPCR_{0.01}\geq 99.5527\%$	$33.2255\%\leq UACI_{0.01}\leq 33.7016\%$
	$NPCR_{0.001}\geq 99.5341\%$	$33.1594\%\leq UACI_{0.001}\leq 33.7677\%$
